# Effects of low-to-moderate ethanol consumption on colonic growth and gene expression in young adult and middle-aged male rats

**DOI:** 10.1371/journal.pone.0243499

**Published:** 2020-12-16

**Authors:** Nicole Wells, Jacqueline Quigley, Jeremy Pascua, Natalie Pinkowski, Lama Almaiman, Susan M. Brasser, Mee Young Hong

**Affiliations:** 1 School of Exercise and Nutritional Sciences, San Diego State University, San Diego, CA, United States of America; 2 Department of Psychology, San Diego State University, San Diego, CA, United States of America; University of Oklahoma Health Sciences Center, UNITED STATES

## Abstract

Excessive alcohol consumption is a risk factor associated with colorectal cancer; however, some epidemiological studies have reported that moderate alcohol consumption may not contribute additional risk or may provide a protective effect reducing colorectal cancer risk. Prior research highlights the importance of proliferation, differentiation, and apoptosis as parameters to consider when evaluating colonic cell growth and tumorigenesis. The present study investigated whether chronic low-to-moderate ethanol consumption altered these parameters of colonic cell growth and expression of related genes. Twenty-four nondeprived young adult (109 days old) and 24 nondeprived middle-aged (420 days old) Wistar rats were randomly assigned to an ethanol-exposed or a water control group (*n* = 12/group). The ethanol group was provided voluntary access to a 20% v/v ethanol solution on alternate days for 13 weeks. Colon tissues were collected for quantitative immunohistochemical analyses of cell proliferation, differentiation and apoptosis using Ki-67, goblet cell and TUNEL, respectively. Gene expression of cyclin D1 (*Ccnd1*), *Cdk2*, *Cdk4*, p21^waf1/cip1^ (*Cdkn1a*), E-cadherin (*Cdh1*) and *p53* were determined by quantitative real-time polymerase chain reaction in colonic scraped mucosa. Ethanol treatment resulted in a lower cell proliferation index and proliferative zone, and lower *Cdk2* expression in both age groups, as well as trends toward lower *Ccnd1* and higher *Cdkn1a* expression. Cell differentiation was modestly but significantly reduced by ethanol treatment only in older animals. Overall, older rats showed decreases in apoptosis and gene expression of *Cdk4*, *Cdh1*, and *p53* compared to younger rats, but there was no observed effect of ethanol exposure on these measures. These findings suggest that low-to-moderate ethanol consumption improves at least one notable parameter in colonic tumorigenesis (cell proliferation) and associated gene expression regardless of age, however, selectively decreased cell differentiation among older subjects.

## Introduction

According to the American Cancer Society, colorectal cancer (CRC) is ranked third in diagnosis and mortality for both men and women, and ranked second in mortality when men and women are combined, with an estimated 147,950 new cases and 53,200 deaths in 2020 [1]. Significant risk factors for colorectal cancer include obesity, lack of physical activity, diets high in red and processed meats, smoking, advanced age, and excessive consumption of alcohol [2,3].

Although excessive alcohol consumption is a known risk factor for CRC development, existing data indicate that lower doses may not have the same impact on risk level. A large-scale study of over a million women conducted in the UK found that low-to-moderate levels of alcohol consumption did not significantly influence risk for cancer development in the colon, however higher intake levels were associated with an increased risk for rectal cancer [4]. Other reports have indicated a nonlinear relationship between ethanol dose and development of CRC, with either no association [5] or protective effects [6,7] at lower doses, while excessive consumption increased risk [7]. These effects may importantly interact with diet, additional risk factors (e.g., obesity), and genetic susceptibility [8]. In humans, moderate or ‘low-risk’ drinking is defined by the National Institute on Alcohol Abuse and Alcoholism and U.S. Department of Health and Human Services Dietary Guidelines for Americans 2015–2020 as up to 1 drink/day for women or 2 drinks/day for men (non-binge levels; <0.08 g/dl blood alcohol concentration) [9]. Data addressing the impact of low-to-moderate ethanol consumption on CRC risk in controlled experimental models is currently very limited.

Prior research has identified cell proliferation, differentiation, and apoptosis as important predictive markers of colonic growth and tumorigenesis [10]. Exposure to carcinogens such as azoxymethane (AOM) increase proliferation and decrease differentiation and apoptosis, while exposure to known chemopreventive diets induce the opposite pattern of effects [11–13]. In C57BL/6 mice, chronic forced ethanol consumption (administered in a Lieber DeCarli diet) has been shown to exacerbate the tumorigenic effects of the carcinogens AOM and dextran sulfate sodium (DSS), increasing both the number and size of colon polyps compared to either carcinogen alone [14]. However, little to no data exists addressing effects of lower levels of ethanol consumption on indices of colonic cell growth, or in the absence of experimentally-induced pathology.

During cell proliferation, cyclin D1 *(Ccnd1)*, cyclin-dependent kinase 2 (*Cdk2*), and cyclin-dependent kinase 4 (*Cdk4*) facilitate progression from the growth to the synthesis phase of the cell cycle [15–17]. This process is frozen at the growth phase through activation of the p53-p21^waf1/cip1^ pathway, which inhibits the activity of *Cdk2* and *Cdk4* [18]. Alternatively, downregulation of *Ccnd1*causes *p21 (Cdkn1a)* to dissociate from *Cdk4* and inhibit *Cdk2*, triggering arrest of the cell cycle independent of *p53* [19]. The primary method by which the *p53* tumor suppressor gene prevents tumorigenesis is through induction of apoptosis [20,21]. Mutations in the *p53* gene usually lead to tumor development, and tumors lacking a functional *p53* gene are often resistant to cancer drug treatment [22]. Therefore, measurement of *p53* levels can provide insight into potential mechanisms of colonic growth and tumorigenesis.

Older adults may show increased sensitivity to ethanol compared to their younger counterparts due to differences in both pharmacokinetic and pharmacodynamic responses to alcohol. The decrease in metabolic rate often observed with aging results in a lower percentage of lean body mass [23], and combined with an increase in body fat percentage and lower body water content, can alter blood alcohol levels and rate of ethanol clearance [24,25]. Furthermore, older adults display increased vulnerability to certain neurobehavioral impairments of alcohol at moderate doses [26], which may extend to other organ systems due to declines in physiological regulatory processes with advancing age [27]. Given significant increased prevalence of alcohol use among older adults over the last two decades [28], comparison of differential health impacts of moderate ethanol consumption between younger and older age groups remains an important area of investigation.

The present study examined the effects of chronic voluntary low-to-moderate ethanol consumption on several measures of colonic cell growth, including cell proliferation, differentiation, and apoptosis, and whether effects of ethanol on these parameters vary as a function of age. We further measured changes in expression of key genes related to these parameters to identify possible mechanisms of colonic growth in response to moderate ethanol treatment.

## Materials and methods

### Animals and diets

This study was conducted in accordance with National Institutes of Health guidelines and was approved by the Institutional Animal Care and Use Committee at San Diego State University. Naive young adult (109 days old) and middle-aged (420 days old) outbred male Wistar rats (n = 24/age) were purchased from Harlan Laboratories (Placentia, CA). Animals at each age were randomly assigned to either an ethanol-exposed or water control group (*n* = 12/treatment group/age). All rats were provided an *ad libitum* regular chow diet (LabDiet, St. Louis, MO, USA) with a macronutrient composition of 28.5% protein, 13.5% fat, and 58% carbohydrates. During experimental procedures, subjects were housed individually in standard plastic-bottom cages in a vivarium that maintained a 12:12-h light/dark cycle and an ambient temperature of approximately 23°C.

### Ethanol exposure

All rats were nondeprived and were exposed for 13 weeks to either a 20% ethanol intermittent-access drinking paradigm [29] or were given access to water alone as their sole fluid source (non-ethanol-exposed control). Animals were initially acclimated to testing procedures for 7 days during which they received free access to food and water only. Following acclimatization, ethanol-exposed rats began 22-h intake sessions involving voluntary access to a 20% v/v ethanol solution vs. water, alternating with 22-h abstinence periods involving voluntary access to water only (45 ethanol drinking sessions total over 13 weeks). The position (right or left) of ethanol and water bottles on each cage was rotated for successive ethanol sessions to control for drinking side preferences. The control group was given unrestricted access to two water bottles on corresponding sides of the cage during the entire duration of the experiment. All fluids were weighed to the nearest gram at the start and end of each session and replenished with fresh fluids daily, and body weights of all rats were measured every 48 h. The 20% ethanol intermittent-access alcohol exposure paradigm has previously been shown to result in mean peak tail blood alcohol concentrations (BACs) of ~30–50 mg/dl (0.03–0.05%) in young adult male Wistar rats within the first two hours of a standard drinking session when BACs are measured at 0.5, 1, 2, 4, and 24 h following onset of ethanol access [29–31]. Prior data from our laboratory has also observed comparable BACs of 28.65 (± 7.23) mg/dl in older male Wistar rats of the same age tested here (14–17 months) when tail blood samples are collected 90 min into the final ethanol drinking session in the intermittent access paradigm [32].

### Sample collection

The day after the final experimental session, rats were euthanized via CO_2_ asphyxiation, and blood and tissue samples were collected for analysis. Blood was centrifuged at 1200×g for 15 min at 4˚C and serum was stored at -80˚C. The colon was opened longitudinally and washed with phosphate buffered saline (PBS), and the colonic mucosa was gently scraped off and frozen and stored at -80˚C. The last 1cm of the distal colon was fixed in 4% paraformaldehyde and embedded in paraffin.

### Colonic cell proliferation measurement

The distal colon sections were deparaffinized in xylene and rehydrated. The sections were then heated in 0.1mM sodium citrate buffer (pH 6.2) for antigen retrieval. Samples were incubated with mouse anti-human Ki-67 antibody (BD Biosciences, San Diego, CA), biotinylated rabbit anti-mouse antibody (Agilent, Santa Clara, CA) and Vectastain ABC Kit (Vector Laboratories, Burlingame, CA) [33]. Samples were stained with DAB (3,3′-diaminobenzidine) then counter-stained with Harris Hematoxylin. Samples were dehydrated and mounted, and the numbers and locations of proliferative cells were recorded. Observers were blind to the treatment and were only given a number for each animal as a reference. For each animal, the number and position of each labelled cell in the crypt column were recorded. There were at least 15 fields examined per animal, then averaged. To calculate proliferation index (PI), the number of proliferative cells was divided by the number of total cells per crypt, then multiplied by 100. To calculate proliferative zone (PZ), the position of the uppermost labeled cell was divided by the total number of crypt cells, then multiplied by 100.

### Colonic cell differentiation measurement

Cell differentiation was measured by goblet cell count, which were identified with alcian blue stain [34]. After deparaffinization and rehydration, samples were stained with alcian blue solution (pH 2.5) (Sigma-Aldrich, St. Louis, MO) for 5 min, then counter-stained with nuclear fast red solution (Sigma-Aldrich) for 5 min. Samples were dehydrated and mounted. The numbers and locations of alcian blue stained cells were recorded. To calculate differentiation index (DI), the number of stained goblet cells was divided by the number of total cells per crypt, then multiplied by 100.

### Colonic cell apoptosis measurement

Apoptotic cells were determined by terminal deoxynucleotidyl transferase (TdT)-mediated dUTP nick end labeling (TUNEL), in order to visualize the 3′-OH ends of DNA fragments found in apoptotic cells [35]. DNA fragments were labeled with TdT (EMD Millipore, Temecula, CA) and bound with anti-digoxigenin antibody. Apoptotic cells were identified, and numbers and locations were recorded. To calculate apoptotic index (AI), the number of apoptotic cells was divided by the number of total cells per crypt, then multiplied by 100.

### RNA extraction and gene expression

Samples were homogenized with Trizol (Invitrogen, Carlsbad, CA) to isolate RNA from colonic mucosa. Chloroform was added, followed by isopropanol, to extract RNA. RNA was washed with ethanol and reconstituted with RNAse-free water. cDNA was synthesized using SuperScript III Reverse Transcriptase (Invitrogen) with oligo(dT)_12–18_ primers.

Gene expression for cyclin D1 (*Ccnd1*), *Cdk2*, *Cdk4*, p21^waf1/cip1^ *(Cdkn1a)*, E-cadherin (*Cdh1*) and *p53* were analyzed using TaqMan probes in quantitative real-time PCR (ViiA7, AppliedBiosystems, Foster City, CA). Each test was performed in duplicate, and results were calculated using ΔΔCT analysis with normalization to r18S expression. One animal from the young control group was used as the control to give a ΔΔCT value, and 18S was not altered by age or treatment.

### Statistical analysis

Ethanol intake in ethanol-exposed rats was measured for each session during the 13-week exposure phase to determine levels and patterns of ethanol consumption across time. Individual session data were averaged in 3-session blocks prior to analysis (15 blocks total). Ethanol intake (g/kg) was analyzed using repeated measures analysis of variance (ANOVA) with block as a within-subject factor, followed by Newman-Keuls test where appropriate (α = 0.05). Body weight, cell proliferation, differentiation, apoptosis, and gene expression were analyzed using one-way ANOVA, two-way ANOVA or student’s t-tests (SPSS Statistics version 24, IBM, Somer, NY). Data were reported as means ± SEs (standard errors). Significance was set at an alpha level of 0.05.

## Results

### Ethanol intake and body weight

**Fig 1:**
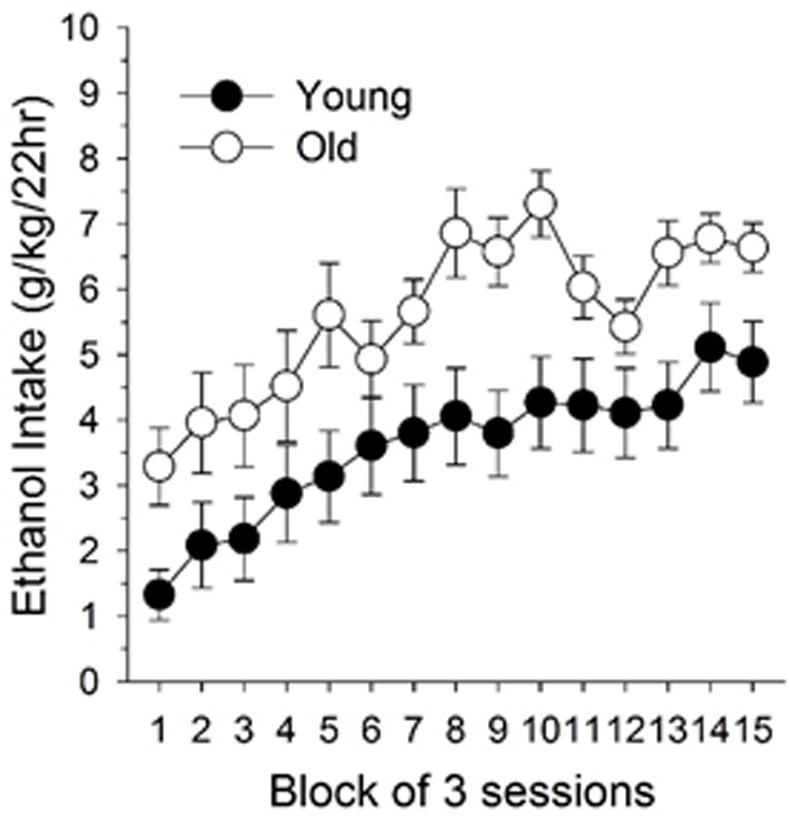
Ethanol intake (g/kg) in young adult (dark circle) and older (clear circle) outbred Wistar rats across all 15 session blocks of ethanol exposure (45 sessions total) in the intermittent access 20% ethanol drinking paradigm. Rats at each age were maintained on a standard chow diet and voluntarily consumed a 20% v/v ethanol solution on alternate days for 13 weeks. The intermittent access procedure resulted in gradually elevated levels of ethanol intake across the first several weeks of ethanol access, followed by stabilization and final average daily ethanol intake values of 4.88 (± 0.62) g/kg in younger rats and 6.64 (± 0.38) g/kg in older rats (effects of session block: F’s_14,154_ > 11.13, P’s < 0.001). Values are expressed as mean ± standard error. Data were analyzed using repeated measures ANOVA, followed by Newman-Keuls post hoc test where appropriate. N = 12/group, total N = 48.

The intermittent ethanol access procedure resulted in gradually elevated ethanol intake levels across the first several weeks of ethanol access followed by stabilization of ethanol intake (effects of session block: *F’s*_14,154_ > 11.13, *P’s* < 0.001), with final mean daily intake values of 4.88 (± 0.62) g/kg in younger rats and 6.64 (± 0.38) g/kg in older rats (Fig 1). For both ages, there were no significant differences in either initial body weight or final body weight between control and ethanol-exposed groups. Weight gain throughout the study also did not differ significantly between ethanol and control treatments for either young adult (Ethanol: 102.00 ± 6.06 g; Control: 110.42 ± 6.89 g) or older rats (Ethanol: 40.67 ± 6.21 g; Control: 54.75 ± 6.14 g).

### Cell proliferation

**Fig 2:**
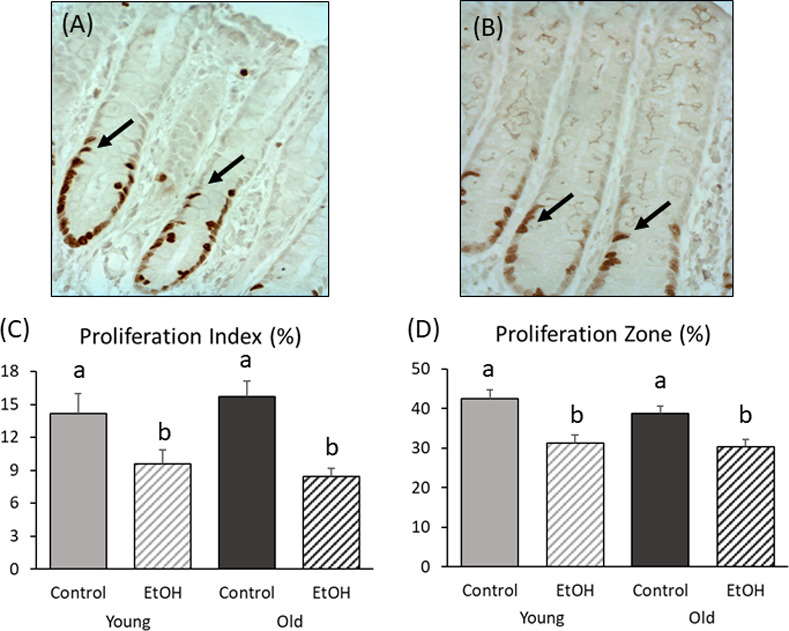
Effect of chronic low-to-moderate ethanol consumption on colonic cell proliferation in young adult and older rats. Adult male Wistar rats were allowed to voluntarily consume a 20% v/v ethanol solution on alternating days for 13 weeks or were given access only to water (non-ethanol-exposed control). This alcohol exposure paradigm results in peak BACs averaging 30–50 mg% in the outbred Wistar strain [29–31]. (A) Cell proliferation (400x) in colonic crypts in older control and (B) older ethanol-exposed. Arrows indicate Ki-67 stained proliferating cells. (C) Ethanol decreased the proliferation index in both young (P = 0.05) and older (P < 0.01) rats, as well as (D) proliferative zone (P < 0.01 for both young and older rats). Bars with different superscripts differ significantly. Data were analyzed using two-way ANOVA, with follow up post hoc t-tests. Young/Control (N = 9); Young/Ethanol (N = 9); Old/Control (N = 9); Old/Ethanol (N = 10).

Cell proliferation was highest in the lower part of colonic crypts (Fig 2A and 2B). Compared to control treatment, cell proliferation index was reduced in ethanol-exposed animals at both younger (P = 0.05) and older (P < 0.001) ages (Fig 2C). The proliferative zone was also decreased in the ethanol group compared to controls for both younger (P = 0.002) and older (P = 0.003) rats (Fig 2D).

### Cell differentiation

**Fig 3:**
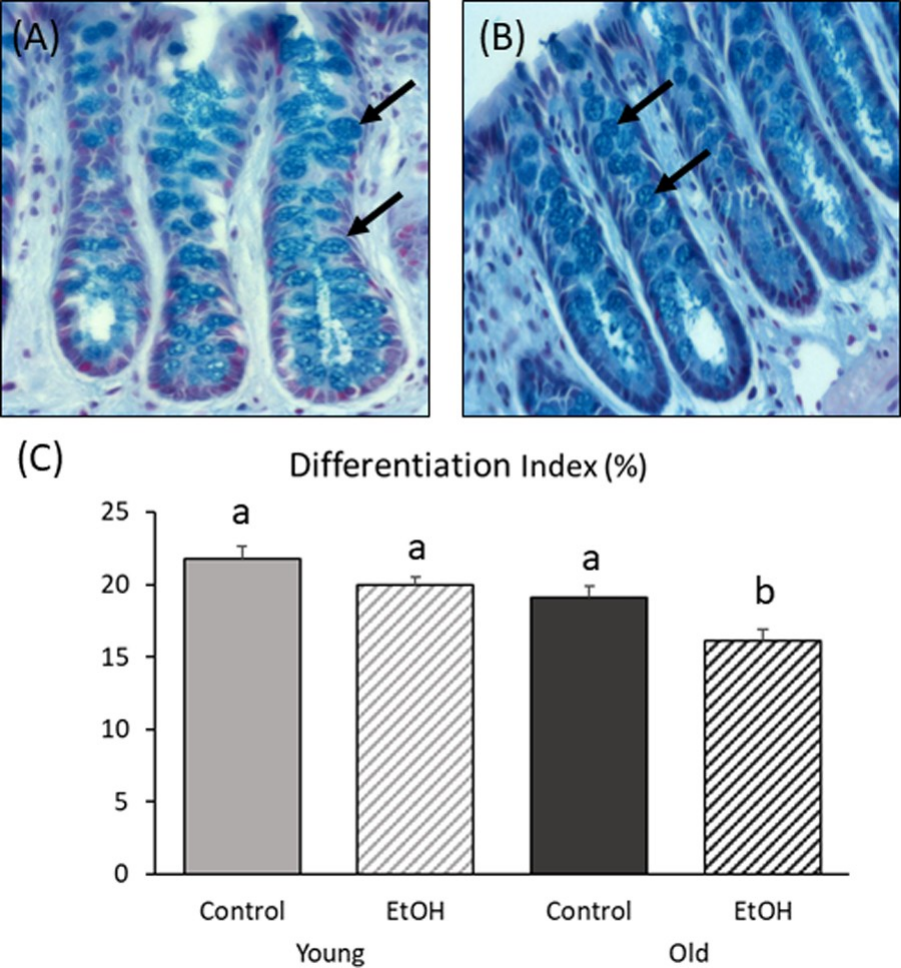
Effect of chronic low-to-moderate ethanol consumption on cell differentiation in young adult and older rats. Adult male Wistar rats at each age were allowed to voluntarily consume a 20% v/v ethanol solution on alternating days for 13 weeks or were given access only to water (non-ethanol-exposed control). (A) Differentiation (400x) in colonic crypts in younger ethanol-exposed and (B) older ethanol-exposed. Arrows indicate alcian stained goblet cells. (C) Ethanol significantly decreased the differentiation index only in older rats (P = 0.001). Bars with different superscripts differ significantly. Data were analyzed using two-way ANOVA, with follow up post hoc t-tests. Young/Control (N = 8); Young/Ethanol (N = 10); Old/Control (N = 11); Old/Ethanol (N = 12).

Differentiation was highest in the middle or upper part of colonic crypts (Fig 3A and 3B). In young adult rats, there was a trend toward lower differentiation in ethanol-treated subjects relative to controls (P = 0.081). In older rats, the differentiation index (DI) was significantly lower in the ethanol-fed group (P = 0.001; Fig 3C).

### Apoptosis

**Fig 4:**
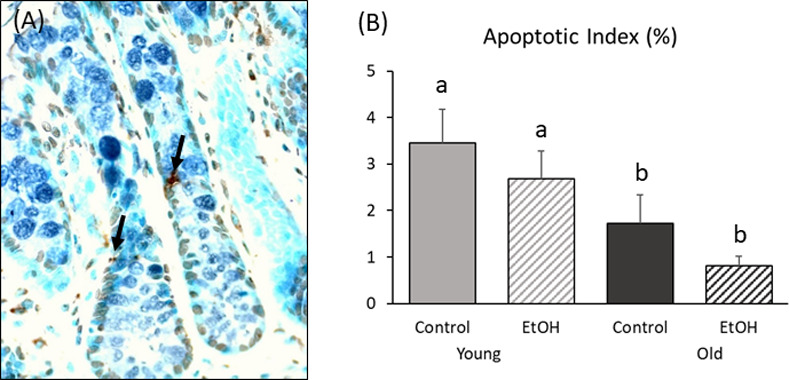
Effect of chronic low-to-moderate ethanol consumption on cell apoptosis in young adult and older rats. Adult male Wistar rats at each age were allowed to voluntarily consume a 20% v/v ethanol solution on alternating days for 13 weeks or were given access only to water (non-ethanol-exposed control). (A) Apoptosis (400x) in colonic crypts of young control. (B) The apoptotic index was lower overall in older rats than in young rats (P < 0.05) but did not differ as a function of ethanol treatment at either age. Bars with different superscripts differ significantly. Data were analyzed using two-way ANOVA, with follow up post hoc t-tests. Young/Control (N = 9); Young/Ethanol (N = 10); Old/Control (N = 10); Old/Ethanol (N = 9).

Colonic apoptotic cells are shown in Fig 4A. There were no significant differences in apoptosis between control and ethanol-exposed animals at either age. However, older rats had a significantly lower apoptotic index (AI) overall than younger rats (P = 0.004; Fig 4B).

### Gene expression

Colonic *Cdk2* gene expression was significantly lower in ethanol-treated rats than in controls (P = 0.017) for both younger and older age groups (Table 1). Cyclin D1 (*Ccnd1*) showed a trend toward lower expression (P = 0.095), and p21^waf1/cip1^ (*Cdkn1a*) showed a trend toward higher expression (P = 0.095) in ethanol-fed rats compared to controls, with no observed effect of age for either gene. Overall, older rats showed significant decreases in *Cdk4* (P = 0.001), E-cadherin (*Cdh1*, P = 0.013), and *p53* (P = 0.005) compared to younger rats, but there were no observed effects of ethanol consumption on these measures. There was no significant interaction between age and treatment for any of the measured genes.

**Table 1:** Expression of genes related to proliferation, differentiation, and apoptosis.

	Young		Old		Significance		
	Control	Ethanol	Control	Ethanol	Age	Treatment	Interaction
***Ccnd1***	2.38 ± 0.41	1.56 ± 0.39	2.33 ± 0.86	1.33 ± 0.41	*0*.*794*	*0*.*095*	*0*.*878*
***Cdk2***	1.62 ± 0.32^a^	0.69 ± 0.14^b^	1.65 ± 0.53^a^	1.02 ± 0.22^b^	*0*.*569*	*0*.*017*	*0*.*633*
***Cdk4***	2.37 ± 0.38^a^	1.80 ± 0.53^a^	0.51 ± 0.10^b^	0.67 ± 0.17^b^	*0*.*001*	*0*.*621*	*0*.*376*
***Cdkn1a***	1.23 ± 0.21	1.27 ± 0.36	0.99 ± 0.22	2.35 ± 0.71	*0*.*305*	*0*.*095*	*0*.*116*
***Cdh1***	1.16 ± 0.15^a^	0.83 ± 0.11^a^	0.52 ± 0.18^b^	0.59 ± 0.23^b^	*0*.*013*	*0*.*450*	*0*.*237*
***p53***	2.37 ± 0.47^a^	1.75 ± 0.46^a^	0.69 ± 0.16^b^	1.01 ± 0.40^b^	*0*.*005*	*0*.*714*	*0*.*260*

Values expressed as means ± standard errors. Numbers with different superscripts differ significantly at P < 0.05. Data were analyzed using two-way ANOVA, with follow up post hoc t-tests. Young/Control (N = 12); Young/Ethanol (N = 12); Old/Control (N = 10); Old/Ethanol (N = 10).

*Ccnd1*: Cyclin D1; *Cdk2*: Cyclin dependent kinase 2; *Cdk4*: Cyclin dependent kinase 4; *Cdkn1a*: p21^waf1/cip1^, *Cdh1*: E-cadherin.

## Discussion

### Cell proliferation

The results of the present study indicate that exposure to low-to-moderate levels of ethanol in a voluntary consumption paradigm improved at least one important parameter of colonic cell growth, resulting in a decrease in colonic cell proliferation for both young adult and middle-aged rats. This intermittent ethanol access procedure produces mean blood alcohol concentrations of ~30–50 mg/dl in young adult Wistar rats when measured within the first two hours of a standard drinking session [29–31] when blood alcohol levels typically peak, and data from our laboratory has measured comparable BACs in this paradigm (~29 mg/dl) in middle-aged Wistar rats between 14–17 months of age. These blood alcohol levels approximate 0.03–0.05% in humans, or that normally resulting from 1–2 standard drinks [36]. The observed reduction in colonic cell proliferation with moderate ethanol treatment is consistent with the effects of chemopreventive agents, which are reported to decrease cell proliferation, and increase differentiation and apoptosis [12,13]. These data are also in line with prior epidemiological studies that have observed an inverse relationship between low-to-moderate levels of ethanol consumption and CRC risk [5–7]. It is critical to note that in contrast, observational data support an elevated risk for colon cancer at higher levels of ethanol consumption [7,37,38] and preclinical findings have also reported an increase in the number of polyps in AOM/DSS mice consuming excessive levels of ethanol, compared to AOM/DSS administration alone [14]. Previous *in vitro* work investigating ethanol effects on cell proliferation in human neoplastic colonocytes has also demonstrated that, while acute levels of ethanol exposure and/or its primary metabolite, acetaldehyde, inhibits cell proliferation, chronic high dose (100 mM) ethanol and acetaldehyde exposure has the opposite effect, enhancing proliferation [39]. Taken together, these data underscore the importance of considering dose-related effects of ethanol exposure on colon, as a majority of studies have examined the impact of excessive concentrations. It is possible that there is a threshold for carcinogenicity of ethanol, which may be importantly modulated by other risk factors, including diet, obesity, genetic vulnerability, and the presence of other carcinogens or existing pathology.

In the current study, *Ccnd1* displayed a trend toward lower expression, and *Cdk2* expression decreased significantly as a function of moderate ethanol treatment, which is consistent with the observed decrease in colonic cell proliferation. Additionally, p21 (*Cdkn1a)*, which inhibits *Ccnd1* and *Cdk2* activity, showed a trend toward higher expression in ethanol-exposed animals regardless of age. Given that *Ccnd1* and *Cdk2* are needed to initiate the synthesis phase of the cell cycle [19], a decrease in expression of these genes should lead to cell cycle arrest and result in decreased proliferation, as observed in the present study. Further, because one function of *Cdkn1a* is to inhibit *Cdk2* activity, an increase in *Cdkn1a* expression would lead to decreased *Cdk2* activity, and therefore lower overall cell proliferation.

Aging increases cell proliferation via acceleration of G1 to S phase transition, as well as progression through the S phase of the cell cycle in the colonic mucosa of aged rats [40,41]. In the latter study, cell proliferation increased in the colonic mucosa of 24-month-old rats, but not in 13-month-old animals, when compared with their younger 4-month-old counterparts. Although our results did not indicate an overall effect of age on cell proliferation, it did not include an advanced age (24-month-old) sample. Examination of an older population may reveal an increase in colonic cell proliferation with advancing age.

### Differentiation

Differentiation, the process by which cells undergo specialization, is decreased in colonic tumorigenesis [10]. The observed reduction in differentiation resulting from moderate ethanol treatment in the current study was significant only among older (middle-aged) subjects, however, there was also a trend for lower colonic cell differentiation in young adult animals. Although modest in size, these effects indicate that ethanol consumption even at low-to-moderate levels may negatively impact cell differentiation and may become more pronounced with increasing age. These findings are consistent with prior *in vitro* evidence that chronic exposure to acetaldehyde, a primary product of ethanol metabolism, decreases differentiation in human colonocytes [39].

Alcian blue-stained goblet cell count is one method for measuring cell differentiation, but it is possible that utilization of an alternative marker could be more sensitive in identifying any effects on colonic differentiation. Other studies examining colonic cancer cell lines have used markers such as alkaline phosphatase [42] or lectin [43,44].

### Apoptosis

The present data demonstrating an overall reduction in colonic apoptosis in middle-aged rats relative to young adult animals concurs with previous findings that apoptosis decreases as a function of age [40]. Apoptosis, a programmed process of cell death, is critical for maintaining colonic tissue homeostasis. Colonic apoptosis is an important mechanism for removal of senescent cells [45]. Inhibition of apoptosis would reduce the clearance rate of senescent cells, which may increase the risk for gene mutation. Colonic apoptosis is inversely associated with CRC risk, so a reduction in apoptosis with age is indicative of elevated CRC risk. The decrease in *p53* expression among older rats in the current study is consistent with the lower level of apoptosis also observed at this age. The *p53* gene is a known tumor suppressor and a key mediator of apoptosis, therefore a reduction in *p53* gene expression should result in a decline in apoptosis [20]. In the present study, there was no observed effect of ethanol consumption on apoptosis or the associated gene *p53*, suggesting that low-to-moderate ethanol consumption does not significantly impact colonic apoptosis. Further research examining ethanol effects on colonic apoptosis could additionally investigate other apoptotic mediators, such as those from the Bcl-2 family, which includes anti-apoptotic proteins like Bcl-2 and pro-apoptotic proteins like Bad, Bax and Bak [46].

Finally, it is important to note that the effects of ethanol ingestion on colonic gene expression and cell kinetics in the present study may be a product of direct effects of ethanol itself, its metabolites, and/or other downstream physiological changes of ethanol ingestion influencing colonic cell regulation. Alcohol is absorbed primarily in the upper intestinal tract and reaches the colon largely from circulating levels in the bloodstream [47]. All tissues in the current study were collected one day after the final alcohol exposure session at a time when ethanol was cleared from blood in order to assess effects of long-term low-to-moderate alcohol exposure, and thus direct levels of alcohol within colon tissue were not assessed. Prior data from our laboratory under the same alcohol exposure conditions in young adult Wistar rats, however, has observed significantly elevated expression of ALDH2 in the colonic mucosa of alcohol-treated rats compared to controls [48], which would allow for enhanced clearance of its primary metabolite acetaldehyde, supporting ethanol metabolism occurring directly within the colon.

## Conclusion

The results of this study contribute to a currently limited literature on the effects of low-to-moderate ethanol intake on several parameters of colonic cell growth and associated gene expression. These findings indicate that low-to-moderate ethanol treatment improved at least one parameter of colonic tumorigenesis, specifically producing a decrease in cell proliferation. This effect was accompanied by altered expression of genes mechanistically involved in proliferation, including a significant decrease in *Cdk2*, and trends toward lower *Ccnd1* and higher *Cdkn1a*. At the same time, there was a modest but significant negative impact of moderate ethanol consumption on cell differentiation, evident among older animals. The present results additionally support previous findings that colonic apoptosis decreases as a function of age, however, was unaffected by low-to-moderate levels of ethanol intake.

The examination of only one sex (males) is a noted limitation of the current study. While overall incidence and mortality rates for colorectal cancer are higher in males than females [49–51], and the association between alcohol intake and CRC risk is stronger in men than in women [51], significant prevalence exists in both sexes. Future studies should include examination of both sexes, including anatomic site-specific differences, as proportion of CRC cases in distal colon is higher in men than in women, while the opposite is observed for cases in proximal colon [49]. Additional research should further examine dose-dependent responses to ethanol on indices of colorectal cancer risk in both younger and older populations, including an extended longitudinal study comparing the effects of chronic low-to-moderate and excessive ethanol consumption, to provide data assessing lifetime risk. It would also be useful to examine an advanced aged population with a higher risk for tumorigenesis, and how differing levels of ethanol intake specifically impact CRC risk in combination with other environmental (e.g., dietary) or genetic risk factors. Finally, continued exploration of concomitant changes in gene expression will provide important insights into specific mechanisms by which ethanol consumption alters parameters of colonic growth, and ultimately influences colorectal cancer risk.

## Supporting information

S1 Appendix Supporting information of data. (PDF)
