# Supplementary material for: Effects of low-to-moderate ethanol consumption on colonic growth and gene expression in young adult and middle-aged male rats
Source: PLoS One. 2020 Dec 16;15(12):e0243499. doi: 10.1371/journal.pone.0243499 (PMC7743962; doi:10.1371/journal.pone.0243499)
Supplement: S1 Appendix — (PDF) [file pone.0243499.s001.pdf]

Ethanol study

Totalcell	TotalPC	BPC	MPC	TPC	Highest	TotalPI	BPI	MPI
30.83	3.11	0.83	0.06	0	11.39	10.09	2.69	0.19
32.9	3.16	1.96	1.2	0	13	9.6	5.96	3.65
31.4	4.1	2.53	1.57	0	12.92	13.06	8.06	5
30.82	4.1	2.53	1.57	0	14.38	13.3	8.21	5.09
Tissue block damaged								
33.5	3.5	2.25	1.25	0	11.41	10.45	6.72	3.73
30.72	4.07	2.97	1.07	0.03	15	13.25	9.67	3.48
30	8	5.5	2.5	0	13.75	26.67	18.33	8.33
29.53	5.41	3.76	1.53	0.12	15.82	18.32	12.73	5.18
30.59	3.9	2.85	1.05	0	11	12.75	9.32	3.43
Tissue block damaged								
Tissue block damaged								
30.62	3.15	2.12	1.03	0	10.56	10.29	6.92	3.36
36.6	0.6	0.6	0	0	8	1.64	1.64	0
30.09	2.36	2.18	0.18	0	6.36	7.84	7.24	0.6
31.48	2.13	1.5	0.63	0	12.2	6.77	4.76	2
29.05	2.88	1.98	0.85	0.05	9.2	9.91	6.82	2.93
28.5	3	2.5	0.5	0	8.78	10.53	8.77	1.75
29	4	4	0	0	9	13.79	13.79	0
30.56	4	2.78	1.22	0	11.33	13.09	9.1	3.99
30.72	3.8	2.84	0.96	0	10.6	12.37	9.24	3.13
Tissue block damaged								

TPI	PZ	DC	DCbottom	DCmiddle	DCtop	DI	DIbottom	DImiddle
0	36.94	7.21	1.08	2.42	3.71	22.5	3.37	7.55
0	39.51	6.64	0.48	2.68	3.48	18.97	1.37	7.66
0	41.15	8	1.64	2.96	3.4	25.71	5.27	9.51
0	46.66	6.99	1.17	2.52	3.39	22.36	3.74	8.06
0	34.06	7.74	1.32	3	3.86	23.58	4.02	9.14
0.1	48.83	7.04	1.04	2.87	3.17	20.21	2.99	8.24
0	45.83							
0.41	53.57	5.74	0.65	2.09	3	18.34	2.08	6.68
0	35.96	6.87	1.43	2.22	3.22	22.64	4.71	7.31
0	34.49	7.42	1.29	2.54	3.58	22.74	3.95	7.78
0	21.86	6.86	0.73	2.64	3.5	19.28	2.05	7.42
0	21.14	6.83	0.83	2.91	3.09	19.73	2.4	8.41
0	38.75	6.91	1.22	2.41	3.27	20.11	3.55	7.01
0.17	31.67	6.59	0.95	2.5	3.14	17.53	2.53	6.65
0	30.81	6.27	0.91	2.45	3	18.49	2.68	7.23
0	31.03	9.05	1.09	3.68	4.27	20.96	2.52	8.52
0	37.07	6.39	1.04	1.96	3.39	18.04	2.94	5.53
0	34.51	6.64	1.32	2.41	2.91	20.18	4.01	7.32
		7.14	1.18	2.41	3.55	22.41	3.7	7.56

DItop	AC	ACbottom	ACmiddle	ACtop	AI	AIbottom	AImiddle	AItop
11.58	0.56	0.48	0.04	0.04	1.92	1.64	0.14	0.14
9.94	2.09	0.14	0.18	1.77	6.25	0.42	0.54	5.29
10.93	1.43	0.04	0.09	1.3	4.61	0.13	0.29	4.19
10.84	0.73	0	0	0.73	2.32	0	0	2.32
11.76	0.05	0	0	0.05	0.14	0	0	0.14
9.1	0.26	0	0	0.26	0.78	0	0	0.78
	1.6	0	0.05	1.55	4.55	0	0.14	4.41
9.58	1.45	0	0	1.45	5.02	0	0	5.02
10.61	1.95	0	0.09	1.77	5.44	0	0.25	4.94
10.97	2.48	0	0.04	2.43	6.66	0	0.11	6.53
9.83	0.41	0	0	0.41	1.06	0	0	1.06
8.93	0.45	0	0	0.45	1.21	0	0	1.21
9.52	0.86	0.45	0	0.82	2.75	1.44	0	2.63
8.35	0.43	0	0	0.43	1.17	0	0	1.17
8.85	1.17	0	0	1.17	4.1	0	0	4.1
9.89	0.36	0	0	0.36	0.86	0	0	0.86
9.57	1.27	0.09	0.18	1	4.2	0.3	0.6	3.31
8.84	0.77	0	0	0.77	1.99	0	0	1.99
11.14	0.91	0	0	0.91	2.91	0	0	2.91

cycliD1colc	CDK2colon	CDK4colon	p21colon	Ecadherinc	p53colon	Totalcell	TotalPC	BPC
1	1	1	1	1	1	31.58	7.75	4.92
1.37	0.81	2.19	0.59	0.84	0.66	30.5	4.06	3.06
1.67	1.2	1.39	0.93	1.24	1.94	Tissue block damaged		
1.64	2.29	4.23	2.17	1.33	4.19	33.3	4.95	3.9
1.37	0.73	3.47	0.73	1.22	1.35	35.38	4.63	2.63
3.1	3.61	2.16	2.59	2.17	3.34	33	7	5
2.75	3.58	1.66	0.34	1.4	5.04	34.89	5.22	4.33
3	1.5	2.34	1.34	0.82	3.15	35.98	4.59	3.61
2.01	0.53	1.16	0.98	0.39	0.66	Tissue block damaged		
1.33	0.34	1.67	0.5	0.52	0.59	Tissue block damaged		
3.29	1.54	1.84	1.39	0.97	1.92	35	4.05	3
6.1	2.36	5.33	2.13	2.03	4.64	37.63	5.63	4.88
2.93	1.75	1.18	1.5	1.15	2.54	37.36	2.55	2.09
1.77	1.3	2.27	1.07	1.14	2.18	33.5	3.56	2.81
1.36	0.71	1.58	0.57	0.88	1.08	27.74	3.52	2.26
0.98	0.34	1.52	3.65	0.82	0.52	33.5	3.22	2.56
1.07	0.6	1.47	0.41	1.06	1.73	39.82	4	3.09
0.5	0.4	1.43	3.46	0.73	5.95	34.1	2.7	2.2
2.31	0.87	1.7	0.57	1.4	1.01	29.5	1.25	1.25
5.07	1.17	7.39	0.79	1.05	1.67	42.73	3.45	2.18
1	0.36	1.46	0.25	0.78	0.76	32.37	2.68	2
0.55	0.23	0.48	0.2	0.28	0.3	39	2.33	1.55
0.95	0.36	0.83	0.27	0.54	0.28	Tissue block damaged		
0.16	0.14	0.24	2.46	0.12	2.98	Tissue block damaged		

MPC	TPC	Highest	TotalPI	BPI	MPI	TPI	PZ	DC
2.75	0.08	15	24.54	15.58	8.71	0.25	47.5	6.86
1	0	9.78	13.31	10.03	3.28	0	32.07	5.1
1.05	0	12.45	14.86	11.71	3.15	0	37.39	6.5
2	0	15	13.09	7.43	5.65	0	42.4	6.72
2	0	14.71	21.21	15.15	6.06	0	44.58	7.36
0.89	0	12.89	14.96	12.41	2.55	0	36.94	6.3
0.98	0	12.22	12.76	10.03	2.72	0	33.96	8
								5.5
								5.42
1.05	0	13.9	11.57	8.57	3	0	39.71	7.96
0.75	0	13.25	14.96	12.97	1.99	0	35.21	6.91
0.46	0	9.73	6.83	5.59	1.23	0	26.04	4.91
0.75	0	10.75	10.63	8.39	2.24	0	32.09	3.05
1.26	0	10.95	12.69	8.15	4.54	0	39.47	5.1
0.66	0	11.54	9.61	7.64	1.97	0	34.45	5.09
0.91	0	12.95	10.05	7.76	2.29	0	32.52	5.56
0.5	0	12.29	7.92	6.45	1.47	0	36.04	4.91
0	0	6.5	4.24	4.24	0	0	22.03	4.7
1.27	0	9.82	8.07	5.1	2.97	0	22.98	3.83
0.68	0	9.08	8.28	6.18	2.1	0	28.05	5.59
0.78	0	11.78	5.97	3.97	2	0	30.21	4.68
								5.45
								4.5

DCbottom	DCmiddle	DCtop	DI	DIbottom	DImiddle	DItop	AC	ACbottom
0.82	2.64	3.41	19.73	2.36	7.59	9.81	1.67	0
0.73	2.05	2.32	14.37	2.06	5.77	6.54	0.36	0
							Tissue block damaged	
0.86	2.32	3.32	19.61	2.6	7	10.02	0.14	0
0.86	2.19	3.67	19.47	2.49	6.34	10.63	0.14	0
0.86	2.77	3.73	19.28	2.25	7.26	9.77	0.59	0
0.94	2.18	3.18	18.21	2.72	6.3	9.19	0	0
1.45	3.14	3.41	22.37	4.05	8.78	9.53	0	0
0.69	2.06	2.75	17.32	2.17	6.49	8.66	0.5	0
0.86	1.91	2.65	16.6	2.63	5.85	8.12	0.41	0
0.87	3.26	3.83	23.84	2.61	9.76	11.47	1.59	0
1	2.82	3.09	19.61	2.84	8	8.77	Tissue block damaged	
0.63	1.73	2.5	15.23	1.95	5.37	7.75	Tissue block damaged	
0.32	1.32	1.41	9.09	0.95	3.93	4.2	0.41	0
0.55	1.55	3	18.61	2.01	5.65	10.94	0.27	0
0.64	1.94	2.64	17.39	2.19	6.63	9.02	0.23	0
0.64	2.16	2.76	16.75	1.93	6.51	8.31	0.27	0
0.68	1.41	2.82	15.26	2.11	4.38	8.76	0.05	0
0.76	1.47	2.47	18.62	3.01	5.82	9.79	0.55	0
0.48	1.26	2.09	13.34	1.67	4.39	7.28	0.23	0
0.82	2.09	2.68	18.33	2.69	6.85	8.79	0.14	0
0.36	1.59	2.72	16.09	1.24	5.47	9.35	0.05	0
0.81	2.81	2.5	18.85	2.8	9.72	8.65	Tissue block damaged	
0.43	1.73	2.32	15.92	1.52	6.12	8.21	Tissue block damaged	

ACmiddle	ACtop	AI	AIbottom	AImiddle	AItop	cycliD1colc	CDK2colon	CDK4colon
0	1.67	5.36	0	0	5.36	1	1	1
0	0.36	0.99	0	0	0.99	1.63	1.21	0.7
						0.38	1.65	0.7
0	0.14	0.42	0	0	0.42	7.97	5.69	0.65
0	0.14	0.43	0	0	0.43	Sample lost		
0	0.59	1.82	0	0	1.82	Sample lost		
0	0	0	0	0	0	0.89	1.27	0.22
0	0	0	0	0	0	6.89	0.19	0.36
0	0.5	1.79	0	0	1.79	1.28	1.8	0.55
0	0.41	1.29	0	0	1.29	1.19	1.85	0.41
0.14	1.45	5.12	0	0.45	4.67	0.52	0.58	0.28
						1.51	1.31	0.21
						3.23	2.64	1.12
0	0.41	1.37	0	0	1.37	0.41	0.37	0.22
0	0.27	1.01	0	0	1.01	Sample lost		
0	0.23	0.68	0	0	0.68	2.15	1.27	1.22
0	0.27	0.83	0	0	0.83	Sample lost		
0	0.05	0.15	0	0	0.15	1.01	0.99	0.4
0	0.55	2.05	0	0	2.05	0.47	0.5	0.07
0	0.23	0.7	0	0	0.7	0.23	0.38	1.06
0	0.14	0.41	0	0	0.41	0.87	1.6	1.12
0	0.05	0.17	0	0	0.17	3.82	1.05	1.04
						0.58	0.61	0.35
						0.56	0.79	0.11

p21colon	Ecadherinc	p53colon
1	1	1
1.56	1.93	1.16
0.28	0.17	0.16
0.15	0.16	0.07
1.74	0.18	0.65
0.14	0.01	0.02
1.31	0.59	1.07
1.99	0.46	1.28
0.53	0.14	0.4
1.23	0.55	1.12
3.25	2.1	4.22
0.28	0.11	0.2
3.93	0.06	1.13
1.05	0.62	1.81
0.61	0.11	0.3
1.27	1.54	1.33
6.79	0.04	0.09
4.94	1.02	0.19
0.57	0.24	0.52
0.78	0.11	0.33
